# Draft genome sequence of *Labrys* sp. strain Lb11, an ʟ-glucose-utilizing bacterium isolated from soil

**DOI:** 10.1128/mra.00099-26

**Published:** 2026-04-20

**Authors:** Yuki Doi, Akito Hama, Akira Nakamura

**Affiliations:** 1 Institute of Life and Environmental Sciences, University of Tsukuba, Tsukuba, Japan; 2 Microbiology Research Center for Sustainability (MiCS), University of Tsukuba 623473, Tsukuba, Japan; 3 Tsukuba Institute for Advanced Research (TIAR), University of Tsukuba 13121, Tsukuba, Japan; Wellesley College 8456, Wellesley, Massachusetts, USA

**Keywords:** ʟ-glucose utilization, *Labrys sp.*, draft genome sequence

## Abstract

We report the draft genome sequence of an ʟ-glucose-utilizing bacterium, *Labrys* sp. strain Lb11, isolated from soil. The genome comprises 7,231,568 bp in 15 contigs, with 6,490 protein-coding genes, 9 rRNA, and 66 tRNA genes. The average nucleotide identity analysis indicates that the strain represents a novel species.

## ANNOUNCEMENT

**Fig 1:**
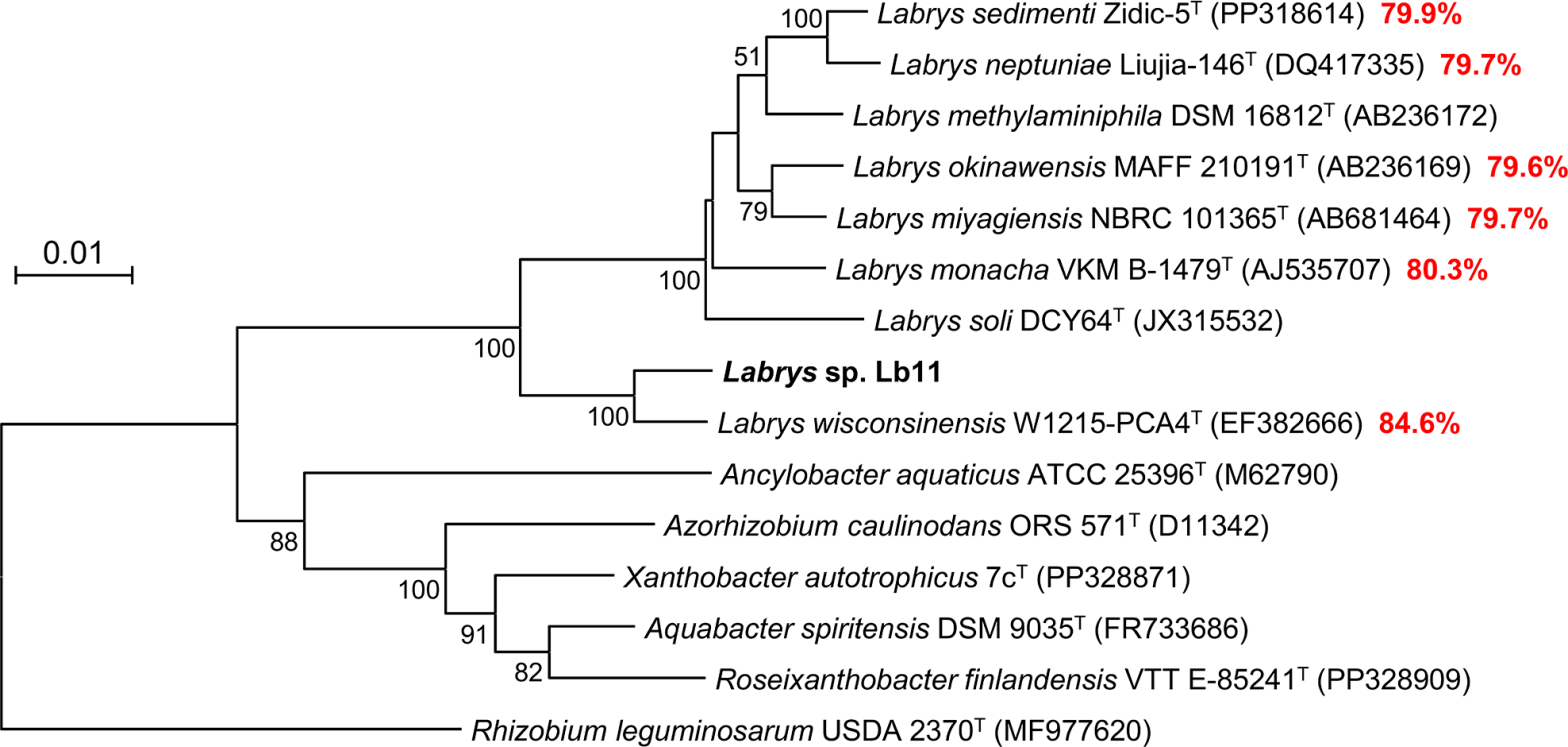
Phylogenetic tree based on 16S rRNA gene sequences of strain Lb11, species of the genus Labrys, and type species of the genera in the family Xanthobacteraceae. *Rhizobium leguminosarum* USDA 2370^T^ was used as an outgroup. The tree was constructed by the neighbor-joining method in MEGA11. Accession numbers are shown in parentheses. Bold numbers in red (%) represent ANI values to strain Lb11.

ʟ-Glucose, the enantiomer of ᴅ-glucose, had been generally considered not to be utilized as a carbon source by living organisms (1); however, we have isolated two ʟ-glucose-utilizing bacterial strains of different phyla, and their catabolic pathways were reported (2, 3). An ʟ-glucose-utilizing strain Lb11 was isolated from a soil of the fertilizer test field in Tsukuba-Plant Innovation Research Center, University of Tsukuba (36° 07′ 07″ N, 140° 05′ 44″ E; Ibaraki, Japan). One milliliter of a soil suspension was added to 100 mL of a minimal medium containing ʟ-glucose (ʟ-GlcMM, 2) and incubated aerobically at 28°C for 4 days. The culture was then spread on ʟ-GlcMM agar plates, and colonies forming after 5 days at 28°C were subcultured on the same medium at least twice to confirm ʟ-glucose utilization. The 16S rRNA gene of strain Lb11 was amplified by colony-PCR using primers 10F (5′-GAGTTTGATCCTGGCTCAG-3′) and 1492R (5′-GGTTACCTTGTTACGACTT-3′), separated by agarose gel electrophoresis, purified using the QIAquick Gel Extraction Kit (QIAGEN, Hilden, Germany), and analyzed using a 3730xl DNA Analyzer (Applied Biosystems, CA, USA). BLAST and phylogenetic analysis using the neighbor-joining method indicated that strain Lb11 belongs to the genus *Labrys* and is closely related to *Labrys wisconsinensis* strain W1215-PCA4^T^ (98.6%) (Fig. 1).

Genomic DNA extracted using Genomic-tip 20G (Qiagen) was purified using DNA Clean Beads (MGI Tech, Shenzhen, China) and sheared to 10–25 kb with Megaruptor 3 (Diagenode, Liege, Belgium) without size selection. The DNA library constructed using the SMRTbell Prep Kit 3.0 and SMRTbell gDNA Sample Amplification kit (PacBio, CA, USA) was sequenced with a Revio system and the Revio polymerase kit (PacBio). High-fidelity reads were generated by SMRT Link v. 25.2.0.266,456 (PacBio). Trimming of PCR adapters and duplicate reads were conducted with lima v. 2.12.0 and pbmarkdup v. 1.0.3 (PacBio), respectively. Short reads (≤1,000 bases) were removed from raw 34,639 reads (average length, 7,148 bp; N50, 8,326 bp) using Filtlong v. 0.2.1 (https://github.com/rrwick/Filtlong), and the remaining 27,458 reads were assembled using Flye v. 2.9.3-b1797 (4). Genomic circularity was confirmed by Bandage v. 0.8.1 (5), and the assembled data were confirmed using CheckM2 v. 1.0.1 (6). Default parameters were used except where otherwise noted.

The draft genome of strain Lb11 comprised 7,231,568 bp assembled into 15 contigs, with an N50 value of 903,871 bp, a GC content of 68.8%, and an average sequencing coverage of 34.2×. Genome completeness and contamination were estimated to be 100% and 7.26%, respectively. Automatic annotation using Prokka v1.14.6 (7) indicated that the contigs contained a total of 6,490 predicted coding sequences (CDSs), 9 rRNA genes, and 66 tRNA genes. The average nucleotide identity (ANI) values between strain Lb11 and *L. wisconsinensis* W1215-PCA4^T^ and *L. monacha* VKM-B1479^T^ were 84.6% and 80.3%, respectively, and the ANI values with the other type strains of this genus were <80% (Fig. 1), indicating that strain Lb11 was not closely related to any currently recognized species (8).

Our study will contribute to investigations of bacterial ʟ-glucose catabolism and expand the known diversity of the genus *Labrys*.

## Data Availability

The 16S rRNA gene sequence accession number for strain Lb11 is LC912710. The draft genome sequence of strain Lb11, assembled into 15 contigs, is available from DDBJ/EMBL/GenBank under the accession number BAAJOT000000000. Raw sequence data were deposited in the DRA database under the accession number DRR899707 (BioProject number PRJDB40113 and BioSample number SAMD01797006).
